# The effect of education on the nursing care quality of patients who are under mechanical ventilation in ICU ward

**DOI:** 10.1016/j.dib.2017.11.090

**Published:** 2017-12-06

**Authors:** Sahar Geravandi, Farhad Soltani, Mohammad Javad Mohammadi, Rashin Alizadeh, Aliasghr Valipour, Abedin Hoseini, Babak Rastegarimehr, Ahmad Reza Yari, Azimeh Karimyan, Ali Ghomeishi

**Affiliations:** a Asadabad school of Medical Sciences, Asadabad, Iran; b Department of Anesthesiology, faculty of medicine, Ahvaz Jundishapur University of Medical Sciences, Ahvaz, Iran; c Abadan school of Medical Sciences, Abadan, Iran; d Department of Health Education and Promotion, School of Health, Tehran University of Medical Sciences, Tehran, Iran; e Research Center for Environmental Pollutants, Qom University of Medical Sciences, Qom, Iran

**Keywords:** ICU, Intensive Care Unit, Patients UMV, Patients Under Mechanical Ventilation, Education, Nurse, Intensive care unit, Mechanical ventilation, Nursing care, Iran

## Abstract

Nurses have the most important role among health care workers (HCWs) in each hospital (Aiken et al., 2008) [1]. Nurses education can lead to the improvement of nursing care If is implemented and designed based on nurses’ needs and proper principles (Aiken et al., 2008) [1]. Nowadays, increased quality of the treatment and increase the chances of survival of patients with acute respiratory failure are very important (Teixeira et al., 2013) [2]. Nursing care plan in ICU patients is one of the important elements in nursing care, and one of the main strategies is promotion of education level. Nurses due to longtime relationship with nursing staff in 24 hours and use of multiple roles of education have excellent position in evaluating educational needs and performing clinical educator roles. The effect of education on the nursing care quality of patients who were under mechanical ventilation (UMV) in intensive care unit (ICU) ward of Razi hospital is evaluated during 2015. The present study is descriptive-analytical and semi experimental research. Research statistical population included 30 nurses. In this paper, the effects of communication with the patient, correct suctioning, compliance of aseptic techniques, the correct discharge of tube cuff, chest physiotherapy, the correct change positions, health food gavage, prevent of foot drop, oral hygiene, the eyes hygiene and protect the cornea have been studied. After completion of the questionnaires by patients, the obtained coded data were fed into EXCEL. Reliability was confirmed with coefficient Alfa 0.86 and the result of software and techniques were entered to SPSS for statistics and analysis.

**Specifications Table**

**Table:** 

Subject area	*Medicine, clinical research*
More specific subject area	*Effect of education on quality of nursing care in ICU ward*
Type of data	*Table, figure*
How data was acquired	*Functional clinical assessment of the patients under mechanical ventilation in intensive care unit and researcher-made questionnaire analysis*
Data format	*Raw, analyzed, Descriptive and statistical data*
Experimental factors	– *Sample consisted of 20 nurses who worked at day/night in ICU wards Razi teaching hospital, Ahvaz.* – *After Inviting the nurses, the researcher-made questionnaire including demographic data and questions which were related to effect of education on quality of nursing care in ICU wards were completed.* – *In this paper, the effects of communication with the patient, correct suctioning, compliance of aseptic techniques, the correct discharge of tube cuff, chest physiotherapy, the correct change positions, health food gavage, prevent of foot drop, oral hygiene and the eyes hygiene and protect the cornea have been studied.*
Experimental features	*Education is one of the most factors* on *quality of nursing care.*
Data source location	*Ahvaz, Iran*
Data accessibility	*Data is included in this article.*

**Value of the data**

• *These data describe factors affecting on nursing care quality in Intensive care unit ward and helps the control and prevention from lack of medical attention.*
• *Due to the importance of the risk factors of Nursing Care in patients who were under mechanical ventilation are discussed in this article.*
• *The results showed that education can be useful for provide better quality of clinical services of patients under mechanical ventilation*
• *Results are also important for patients under mechanical ventilation admitted to ICU ward.*
• *Based on research findings, one of the most important factors of providing better quality of clinical services of patients under mechanical ventilation has been obtained.*

## 1 Data

Table 1 represents demographic characteristics of nurses in ICU wards of Razi teaching hospital of Ahvaz, Iran during 2015 which are used for description of experiments. Based on the results of Table 1, the mean age of participants was 32±3.54 years and most of them are women. According to findings of this study, 30% of them had lower than 1–5 years job experience and 70% had 5 years and more. Table 2, Table 3 show the factors ranking on nursing care quality of patients who were under mechanical ventilation in ICU ward of Razi hospital before and after education. According to result, significant differences (*P*<0.05) were observed in communication with patient (*P*=0.024), correct suctioning (*P*=0.001), compliance of aseptic techniques (*P*=0.003), the correct discharge of tube cuff (*P*=0.002), chest physiotherapy (*P*=0.001), the correct change positions (*P*=0.031), health food gavage (P=0.002), Back rub (*P*=0.042), prevent of foot drop (*P*=0.035), oral hygiene (*P*=0.002), and the eyes hygiene and protect the cornea (*P*=0.003) had significant differences. The results of this study showed that the amount of knowledge and great attitude of nurses in nursing care quality of patients who were under mechanical ventilation in ICU ward was 75% before the assessment and education, and after educational programs to 89% (*P*=0/002). Findings also showed that education and participate in educational classes of this center will increase 14% of nurses awareness in nursing care quality of patients under mechanical ventilation in ICU ward (*P*=0/003).

**Table 1:** Characteristics of the nurses in ICU wards of Razi teaching hospital.

**Parameter**	**Characteristics**	**Number (*n*)**	**Percent (%)**
**Age**	Less than 25	3	15
	25–35	10	50
	35 years and more	7	35
			
**Gender**	Female	20	100
	Male	0	–
			
**Years of work experience**	1–5 years	6	30
	5 years and more	14	70
			
**Education level**	Master of sciences and more	2	10
	Bachelor's degree	18	90

**Table 2:** Ranking the factors on quality of nursing care of patients under mechanical ventilation in ICU ward in Razi Hospital before education.

**Basic care from patients under mechanical ventilation**	**Scale (Agree) F (%)**				
	Strongly agree	Agree	Neutral	Disagree	Strongly disagree
Communication with the patient	10(50%)	2(10%)	5(25%)	2(10%)	1(5%)
Suctioning correctly	9(45%)	6(30%)	3(15%)	1(5%)	1(5%)
Compliance of aseptic techniques	12(60%)	3(15%)	2(10%)	2(10%)	1(5%)
The correct discharge tube cuff	10(50%)	3(15%)	3(15%)	3(15%)	1(5%)
Chest physiotherapy	8(40%)	6(30%)	3(15%)	2(10%)	1(5%)
The correct change positions	10(50%)	4(20%)	3(15%)	2(10%)	1(5%)
Health food gavage	12(60%)	3(15%)	2(10%)	2(10%)	1(5%)
Back rub	11(55%)	3(15%)	1(5%)	2(10%)	3(15%)
Prevent of foot drop	9(45%)	4(20%)	1(5%)	3(15%)	3(15%)
Oral hygiene	12(60%)	3(15%)	1(5%)	2(10%)	2(10%)
The eyes hygiene and protect the cornea	13(65%)	2(10%)	1(5%)	2(10%)	2(10%)

**Table 3:** Ranking the factors on quality of nursing care of patients under mechanical ventilation in ICU ward in Razi Hospital after education.

**Basic care from patients under mechanical ventilation**	**Scale (Agree) F (%)**					**P value**
	Strongly agree	Agree	Neutral	Disagree	Strongly disagree	
Communication with the patient	12(65%)	3(15%)	2(10%)	1(5%)	1(5%)	0.024
Suctioning correctly	15(75%)	2(10%)	1(5%)	1(5%)	1(5%)	0.001
Compliance of aseptic techniques	14(70%)	2(10%)	3(15%)	1(5%)	0(0%)	0.003
The correct discharge tube cuff	12(60%)	3(15%)	3(15%)	1(5%)	1(5%)	0.002
Chest physiotherapy	13(65%)	2(10%)	2(10%)	3(15%)	0(0%)	0.001
The correct Change Positions	14(70%)	3(15%)	2(10%)	0(0%)	1(5%)	0.031
Health food gavage	14(70%)	2(10%)	2(10%)	1(5%)	1(5%)	0.002
Back rub	16(80%)	1(5%)	1(5%)	1(5%)	1(5%)	0.042
Prevent of foot drop	14(70%)	4(20%)	1(5%)	1(5%)	0(0%)	0.035
Oral hygiene	15(75%)	2(10%)	2(10%)	1(5%)	0(0%)	0.002
The eyes hygiene and protect the cornea	15(75%)	3(15%)	2(10%)	0(0%)	0(0%)	0.003

## 2 Experimental design, materials and methods

### 2.1 Study area description

**Fig. 1:**
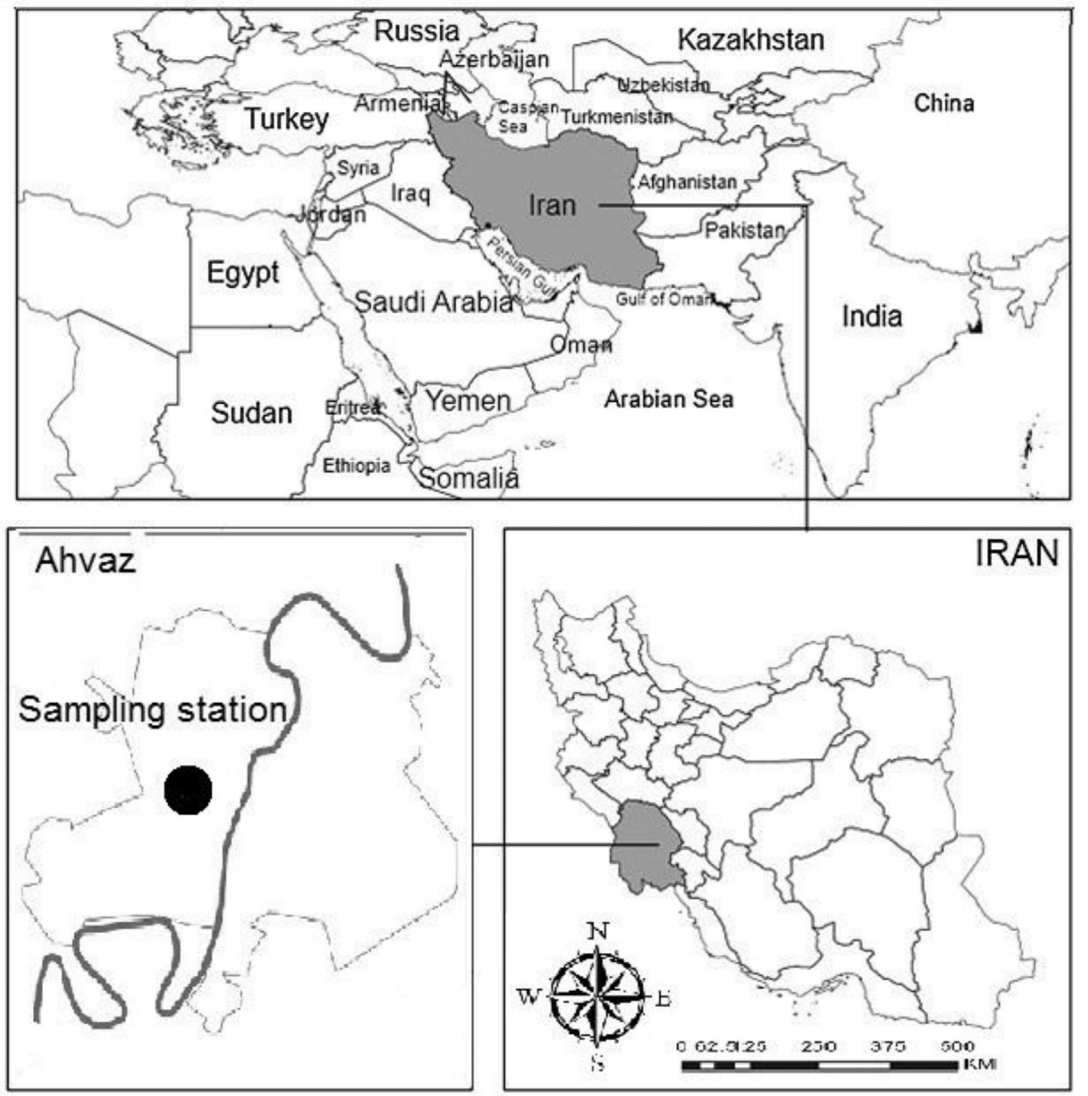
Location of the study area Razi Hospital, in the Southwestern Ahvaz city.

This semi-experimental study was performed at Razi teaching hospital of Ahvaz (principle referral center for infectious diseases in Khuzestan province of Iran) [3]. Khuzestan is one of the most province hot in Iran [4]. Location of the study was in the Ahvaz city which is between 48° and 49°29′ east of the Greenwich meridian, 31°and 45′ minutes north of the equator [5], [6], [7]. Razi teaching hospital with 220 beds approximately is Located in the central of Ahvaz in the southwest of Iran (see Fig. 1).

### 2.2 Experimental design, materials and methods

The target population comprised 20 nurses who were worked at day/night in ICU wards. Data were collected during 2015, including demographic information (characteristics such as age, sex and experience) and questions which were related to the effect of education on nursing care quality in ICU wards of the hospital. In this study, the supervisor, according to observation and after consultation with anesthesiologist, was recorded the result. The Lesson content was included the care of patients who were under mechanical ventilation. The checklist was included 20 nursing care items in intensive care units and was completed one month before and after education with perception by researcher [1], [2], [8], [9]. Nurses who were working in ICU wards of the hospital selected by consensus method in this study. Then, the collected data were coded and entered into SPSS version 16. Data analysis was performed, using SPSS-16. All risk factors were analyzed. The data were analyzed, applying descriptive and statistical tests including independent *t*-test and chi-square. P value was considered significant when *p*<0.05. The significance level in this study was determined 0.05=*α*.
