# Supplementary material for: The effect of education on the nursing care quality of patients who are under mechanical ventilation in ICU ward
Source: Data Brief. 2017 Dec 6;16:822–7. doi: 10.1016/j.dib.2017.11.090 (PMC5773446; doi:10.1016/j.dib.2017.11.090)
Supplement: Supplementary file 1 — Supplementary material [file mmc1.doc]

**The Effect of Education on the Nursing Care Quality of Patients who are under Mechanical Ventilation in ICU ward**

**Conflicts of Interest**

Authors have no conflicts of interest.

**Acknowledgment**

The authors would like to thank student Research committee, Ahvaz Jundishapur University of Medical Sciences for providing financial supported by the grant: (94s17) of this research.

**Funding/Support**

The authors would like to thank student Research committee, Ahvaz Jundishapur University of Medical Sciences for providing financial supported by the grant: (94s17) of this research.
